# Patient safety culture in the operating room: a cross-sectional study using the Hospital Survey on Patient Safety Culture (HSOPSC) Instrument

**DOI:** 10.1186/s12913-022-08756-y

**Published:** 2022-11-29

**Authors:** Arinze D.G. Nwosu, Edmund Ossai, Francis Ahaotu, Okechukwu Onwuasoigwe, Adaobi Amucheazi, Irene Akhideno

**Affiliations:** 1 Department of Anaesthesia, National Orthopaedic Hospital, Enugu, Nigeria; 2 Department of Community Medicine, College of Health Sciences, Ebonyi State University, Abakaliki, Nigeria; 3 Department of Orthopaedics, National Orthopaedic Hospital, Enugu, Nigeria; 4 Department of Orthopaedics, University of Nigeria, Nsukka, Enugu State Nigeria; 5 Department of Anaesthesia, University of Nigeria, Nsukka, Enugu State Nigeria; 6 Department of Anaesthesia, Irrua Specialist Teaching Hospital, Irrua, Edo state Nigeria

**Keywords:** Operating rooms, Organizational culture, Perception, Safety management, Surgery

## Abstract

**Background:**

Credible evidence has established a link between the level of patient safety culture in healthcare environments and patient outcomes. Patient safety culture in the operating room has received scant attention despite the burden of adverse events among surgical patients. We aimed to evaluate the safety culture in our operating rooms and compare with existing data from other operating room settings.

**Methods:**

We investigated the patient safety culture in the operating rooms of our hospital as perceived by the surgeons, nurse anaesthetists and perioperative nurses using the Hospital Survey on Patient Safety Culture (HSOPSC) instrument. IBM Statistical Package for Social Science software, version 25, was used for data entry and analysis. Differences were considered significant when p < 0.05.

**Results:**

Only 122 completed surveys were returned out of a survey population of 132 frontline staff, yielding a response rate of 92.4%. The overall average composite score was 47%. The average composite scores ranged from 17–79.6% across the 12 dimensions of the HSOPSC, with *teamwork within units* being the only dimension with demonstrable strength. *Non-punitive response to error*, *communication openness*, *feedback and communication about error*”, *frequency of events reported*”, *handoffs and transition* and *staffing* need improvement. The perceived safety culture varied according to work areas and professional roles with nurse anaesthetists having the highest perception and the surgeons the least.

**Conclusion:**

Patient safety culture in our operating rooms is adjudged to be weak, with only one of the twelve dimensions of HSOPSC demonstrating strength. This is notwithstanding its comparative strengths relative to other operating room settings.

## Introduction

Two decades ago, while adopting the resolution on patient safety in healthcare at the 55th World Health Assembly the World Health Organization (WHO) recognized the need to promote patient safety as a fundamental principle of all health systems and urged support for member states to promote a culture of safety within health care organizations and encourage research into patient safety [1]. But unsafe care has remained a major source of morbidity and mortality [2], prompting the WHO to launch the first World Patient Safety Day on 17 September, 2019. Beyond the ethical issues of patients suffering personal harm while undergoing care, the burden on the health system encompasses additional treatment, prolonged hospital length of stay, disability and deaths. Very profound too, is the associated phenomena of ‘second’ ‘third’ and ‘fourth’ victims; representing further impact on the involved healthcare professionals, the hospital reputation, and patients who may be harmed subsequently, respectively [3]. Reports from the Irish National Adverse Events Study (INAES) indicate that about 7% of the healthcare adverse events contribute to death, while as much as 70% of these events were considered preventable [4]. Surgical procedures probably account for the majority of healthcare adverse events [5–7]. Recent global estimates suggest that over 7 million people suffer surgical complications annually, with over 1 million deaths [8].

Patient safety culture is a product of individual and group values, attitudes, perceptions and competencies that determine a pattern of behavior and commitment to the safety of patients. Despite the established link between the level of patient safety culture in healthcare environments and patient outcomes [9–11], safety culture in the operating room (OR) has not received significant attention. Unfortunately too, the few available studies suggest that poor safety culture in the OR is pervasive. In one multicentre study poor scores were reported in all the 10 dimensions (composites) of patient safety culture evaluated [12]. While not losing sight of the numerous studies on other work areas in the hospital, the variability of safety culture even across units within a hospital make extrapolations with these other settings untenable [13].

The increasing interest in patient safety over the past two decades has led to the development of several tools for assessing safety culture and climate in healthcare. Among these the Hospital Survey on Patient Safety Culture (HSOPSC) [14] and the Safety Attitudes Questionnaire (SAQ) [15] are the most utilized. A direct comparison of the SAQ and the HSOPSC by simultaneous administration of both tools on healthcare workers (HCWs) had concluded that the reliability of both instruments showed marked similarity [16]. However, based on the analysis conducted on several safety culture assessment tools, the HSOPSC was adjudged to be the most psychometrically sound, by Fin et al. [17].

Earlier large scale studies in healthcare organizations indicate that frontline personnel’s perceptions of better safety climate were superior to management’s perceptions in predicting the risk of adverse outcomes [18, 19]. The perception of frontline personnel regarding the safety culture in the OR could therefore present a reliable basis for evaluating and improving the safety of surgical patients.

Our objective was to assess the current state of patient safety culture in the OR of our hospital, identifying strengths and areas that require improvement. We also sought to compare our safety culture with OR settings elsewhere. This process is in tandem with the Council of Europe’s recommendation on management of patient safety, to the effect that defining the existing safety culture in the organization is the first stage in developing a safety culture [20].

## Methods

This is a cross-sectional descriptive paper-based survey of a purposive sample of frontline operating room personnel in a regional trauma and burns centre.

### Study setting

The 400-bed referral centre which was established in 1973 serves a population of about 80 million encompassing the Southeast where it is located, but also the South-south and North-central geopolitical zones of Nigeria. It is one of the three tertiary hospitals in the city, located within 10 min drive from the Akanu Ibiam international airport, Enugu. The hospital also provides care to a substantial number of secondary, and even primary care patients. There are six operating theatres on-site, at different locations. The surgical specialties include trauma, orthopaedics, spine, plastic and reconstructive surgery, and burns care. Our OR personnel typically consists of; the resident surgeons (with, or without the consultant surgeon), the nurse anaesthetist (with, or without the consultant anaesthetist), the perioperative/circulating nurse, the operating room attendant, and sometimes the radiographer. Anaesthesia service in the institution is essentially nurse-based, with one resident doctor and three consultant anaesthetists.

### Survey Tool

The Hospital Survey on Patient Safety Culture (HSOPSC)/ SOPS® Hospital Survey [14].

The HSOPSC was designed by the United States Agency of Healthcare Research and Quality (AHRQ) in 2004, for the purpose of measuring patient safety culture in individual health institutions [14]. It is a self-reported tool designed specifically for HCWs, requesting for their opinions about the culture of patient safety at their hospitals. It proposes the assessment of 12 dimensions/composite measures pertaining to the climate of patient safety in hospital setting. The culture of safety is measured from the staff perspective.

The HSOPSC consists of 42 items distributed among the 12 dimensions namely; *overall perception of patient safety*, *teamwork within units*, *teamwork across units*, *supervisor/manager expectations and actions promoting safety*, *organizational learning-continuous improvement*, *executive management support for patient safety*, *feedback and communication about error*, *communication openness*, *frequency of error reporting*, *staffing*, *handoffs and transitions between units and shifts* and *non-punitive response to error*. The answers to the items are scaled 1 to 5; 1 and 2 were considered negative towards patient safety, 3 was considered neutral and answer 4 and 5 were considered positive towards patient safety. Out of the 42 items, 17 were negatively framed for psychometric balancing; with the answers reverse-scored prior to recoding into positive, neutral or negative. In effect whereas “agree” and “strongly agree” are ordinarily positive responses, in the negatively framed items “disagree” and “strongly disagree” represent the positive responses. In sum higher values always indicate better perceived safety culture. The composite scores were expressed as the mean percentage of positive answers in the items within each dimension/composite. The overall average composite score was determined as the average of the 12 composite scores. In addition to the 42 items there were also one item each on the respondent’s perception of safety quality in their respective work area and the number of adverse events they have reported in the past 12 months. Six other items sought information regarding the respondent’s service background.

The English version of the survey instrument; SOPS® Hospital Survey Version 1.0. [14] which was obtained online was used for the paper survey.

### Modification of the instrument

A minor modification was deemed necessary in order to facilitate effective communication and comprehension of the item in our cultural background. This was effected in Section F, item 3: “Things fall between the cracks” when transferring patients from one unit to another was changed to… “things escape attention” when transferring patients from one unit to another’, as the former is an unfamiliar phrase in our environment. Such minor modification which will minimally impact on the psychometric properties of the instrument is in compliance with the instrument guideline [14].

### Inclusion criteria

All operating room frontline personnel (attending surgeons, resident surgeons, nurse anaesthetists and perioperative nurses) who have been in active clinical service in the hospital for a period of not less than six months were eligible to participate in the study.

### Exclusion criteria

Eligible but non-consenting OR personnel were excluded. New employees who had spent less than 6 months in the hospital employment were deemed ineligible and consequently excluded.

### Sampling method and respondent selection

The questionnaires were distributed to the entire population of eligible OR frontline personnel who consented to participate in the survey. The sparse number of physician anaesthetists and radiographers precluded them from consideration in the survey in line with the instrument guideline [14]. This recommendation was in consideration of the need to protect the confidentiality of the respondents. On the other hand, the operating room attendants in our setting who are less well-educated had considerable difficulty comprehending the instrument items during pretesting of the instrument and were thus excluded.

### Study procedure

The eligible respondents were invited to participate in the survey in the understanding that their participation is voluntary and that they are at liberty to withdraw their consent at any stage, if they so wish. The paper survey instrument was distributed in-person by the research assistant to all the consenting HCWs at their duty posts for self-administration. The questionnaires were anonymized by leaving no identification code and distributing them enclosed in brown envelopes. Each respondent was urged not to discuss their responses with other staff while further assuring them that their responses will be kept confidential. In order to enhance capture, the nominal role obtained for each of the relevant units was used to tick off the respective staff during distribution and collection of the questionnaires. The questionnaire administration lasted until all those who were absent during the initial phases of the distribution on account of shift duty, or short periods of leave, were captured. Due to the busy schedule of duty the questionnaires were left with the staff to fill in at their soonest convenient time, while the research assistant made repeat visits to further distribute and recover the filled questionnaires. Each of the three groups of HCWs was surveyed sequentially, and the returned surveys marked with a group identifier to ensure that no false claim of group identity occurs. This is then followed the by another group; in the sequence; nurse anaesthetists, perioperative nurses, and surgeons. The survey was conducted from February 7, 2022 to March 11, 2022.

### Data management

This paper survey did not use individual identifiers. Instead, group identifiers were marked on the returned surveys of each of the three categories of HCWs. Later, all the completed paper surveys were marked with identification numbers to serve as respondent identifier but without any information linking the identifiers to individual worker. The respondent and group identifiers were reflected as such in the electronic data file entry. All data entry was accomplished using the IBM SPSS version 25 statistical package. The obtained data were illustrated using tables and bar charts. The percent positive scores for each item of safety culture, as well as the composite scores were computed from the scaled responses of the HCWs. For each item, or composite measure, percentages greater than 75% are considered as strengths and deemed to have positive perception of patient safety culture, while those ≤ 50% indicate weak perceptions of safety culture and require improvement [21].

Comparison of mean scores between the three study groups was done using One-way analysis of variance (ANOVA) test while inter group comparisons were done using Tukey HSD post hoc test. A difference was considered significant when p < 0.05.

### Research Ethics

The study protocol for the survey was reviewed and approved by the Research Ethics Committee of National Orthopaedic Hospital, Enugu. (IRB Number S.313/IV/; Protocol Number 2022/1/103). Only consenting eligible HCWs were recruited, having duly signed to a written informed consent form.

## Results

The derived overall average composite score of patient safety culture was 47%. Out of the 132 eligible and consenting personnel that were invited to the survey, 122 surveys were returned yielding a response rate of 92.4% (122/132). Only three eligible OR personnel were not invited; one on account of maternity leave while two others were on annual leave. None of the returned surveys was excluded in the analysis as they were duly completed and deemed eligible, save for the few items that some respondents did not oblige a response. Twenty six surveys (21%) did not have complete answers to all the items but were utilized in the analysis. The incomplete surveys mostly have only one missing answer, but one survey had as many as 28 missing answers. Where the respondents marked two answers for one item, such inappropriate response is treated as missing/no response. Complete responses were provided in 96 (79%) of the surveys.

One hundred and twenty two (122) HCWs; comprising consultant surgeons (11), resident surgeons (48), nurse anaesthetists (27) and perioperative nurses (36) participated in the study. Over 93% of the respondents have worked in the hospital for more than a year, while 78.7% have worked in their current unit for more than a year and 95.1% have worked in their current specialty for more than one year. About 11% of the respondents reported working for more than 100 h per week (Table 1).

**Table 1:** Background information of the respondents

Variable	Frequency (n = 122)	Percent (%)
**Hospital unit**		
Anesthesiology	27	22.1
Surgery	59	48.4
Peri-operative nursing	36	29.5
**Duration of work in the hospital**		
< 1 year	8	6.6
1–5 years	44	36.1
6–10 years	24	19.7
11–15 years	28	23.0
16–20 years	5	4.1
≥ 21 years	13	10.7
**Duration of work in current hospital unit**		
< 1 year	26	21.3
1–5 years	42	34.4
6–10 years	22	18.0
11–15 years	22	18.0
16–20 years	4	3.3
≥ 21 years	6	4.9
**Number of hours per week you work in the hospital**		
20–39 h	13	10.7
40–59 h	51	41.8
60–79 h	29	23.8
80–99 h	16	13.1
≥ 100 h	13	10.7
**Staff position in hospital**		
Registered nurse	63	51.6
Resident surgeon	48	39.3
Consultant surgeon	11	9.0
**Duration of work in current specialty**		
< 1 year	6	4.9
1–5 years	34	27.9
6–10 years	26	21.3
11–15 years	35	28.7
16–20 years	14	11.5
≥ 21 years	7	5.7

The item with the highest percentage positive response was “People support one another in this unit” (91.8%) while “Staff feel free to question the decisions or actions of those with some authority” received the lowest percentage positive response (10.2%); (Table 2). Only five (A1, A3, A4, A6, B4) out of the 42 items of the HSOPSC instrument were perceived as having ‘strength’ regarding safety culture by the OR personnel (Table 2).

**Table 2:** The 42 items scores

Variable	Negative (%)	Neutral (%)	Positive (%)	Remarks/Recommendation
A1 People support one another in this unit	8 (6.6)	2 (1.6)	112 (91.8)	Strength
A2 We have enough staff to handle the workload	103 (84.4)	2 (1.6)	17 (14.0)	Needs improvement
A3 When a lot of work needs to be done quickly, we work together as a team to get the work done	12 (9.9)	7 (5.7)	103 (84.4)	Strength
A4 In this unit, we treat each other with respect	12 (9.8)	18 (14.8)	92 (75.5)	Strength
A5 Staff in this unit work longer hours than is best for patient care	93 (76.8)	14 (11.6)	14 (11.6)	Needs improvement
A6 We are actively doing things to improve patient safety	8 (6.7)	13 (10.7)	100 (82.6)	Strength
A7 We use more temporary staff than is best for patient care	16 (23.3)	22 (18.2)	83 (68.6)	
A8 Staff feel that their mistakes are held against them	77 (63.7)	22 (18.2)	22 (18.2)	Needs improvement
A9 Mistakes have led to positive changes here	30 (15.2)	27 (22.7)	62 (52.1)	
A10 It is just by chance that more serious mistakes don’t happen around here	38 (31.6)	15 (12.5)	67 (55.8)	
A11 When one area in this unit gets easily busy, others help out	26 (21.5)	14 (11.6)	81 (66.9)	
A12 When an event is reported, it feels like the person is being written up, not the problem	75 (63.6)	21 (17.8)	22 (18.6)	Needs improvement
A13 After we made changes to improve patient safety, we evaluate their effectiveness	21 (17.5)	30 (25.0)	69 (57.5)	
A14 We work in ‘crisis mode’ trying to do too much, too quickly	71 (59.2)	25 (20.8)	24 (20.0)	Needs improvement
A15 Patient safety is never sacrificed to get more work done	44 (37.0)	13 (10.90)	62 (52.1)	
A16Staff worry that mistakes they make are kept in their personnel file	79 (65.8)	24 (20.0)	17 (14.2)	Needs improvement
A17 We have patient safety problems in this unit	38 (31.7)	27 (22.5)	55 (45.8)	Needs improvement
A18 Our procedures and systems are good at preventing errors from happening	30 (24.8)	27 (22.3)	64 (52.9)	
B1 My supervisor says a good word when he/she sees a job done according to established pattern safety procedures	15 (12.3)	17 (13.9)	90 (73.8)	
B2 My supervisor seriously considers staff suggestions for improving patient safety	19 (15.7)	17 (14.0)	85 (70.3)	
B3 Whenever pressure builds up, my supervisor wants us to work faster even if it means taking shortcuts	34 (27.9)	29 (23.8)	59 (48.4)	Needs improvement
B4 My supervisor overlooks patient safety problems that happen over and over	9 (7.4)	14 (11.5)	99 (81.2)	Strength
C1 We are given feedback about changes put into place based on event reports	41 (33.9)	47 (38.8)	33 (27.2)	Needs improvement
C2 Staff will freely speak up if they see something that may negatively affect patient care	18 (25.0)	43 (35.8)	59 (49.2)	Needs improvement
C3 We are informed about errors that happen in this unit	17 (14.1)	41 (33.9)	63 (42.0)	Needs improvement
C4 Staff feel free to question the decisions or actions of those with some authority	86 (72.9)	20 (16.9)	12 (10.2)	Needs improvement
C5 In this unit, we discuss ways to prevent errors from happening	21 (18.0)	27 (23.1)	69 (59.0)	
C6 Staff are afraid to ask questions when something does not seem right	48 (40.3)	44 (37.0)	27 (22.7)	Needs improvement
D1 When a mistake is made but is *caught and corrected before affecting the patient*, how often is this reported?	50 (41.3)	39 (32.2)	32 (26.4)	Needs improvement
D2 When a mistake is made but has *no potential to harm* the patient, how often is this reported?	62 (51.2)	39 (32.2)	20 (16.6)	Needs improvement
D3 When a mistake is made, that *could harm the patient* but does not, how often is this reported?	41 (34.1)	41 (34.2)	38 (31.6)	Needs improvement
F1 Hospital management provides a work climate that promotes patient safety	35 (28.7)	27 (22.1)	60 (49.2)	Needs improvement
F2 Hospital units do not coordinate well with each other	48 (39.4)	20 (16.4)	54 (44.3)	Needs improvement
F3 Things escape attention when transferring patients from one unit to another	39 (31.9)	32 (26.2)	51 (41.8)	Needs improvement
F4 There is poor cooperation among hospital units that need to work together	26 (21.4)	23 (18.9)	73 (59.8)	
F5 Important patient care information is often lost during shift changes	41 (24.2)	18 (15.0)	61 (50.8)	
F6 It is often unpleasant to work with staff from other hospital units	23 (19.2)	26 (21.7)	71 (59.1)	
F7 Problems often occur in the exchange of information across hospital units	42 (35.0)	25 (20.8)	53 (44.2)	Needs improvement
F8The actions of hospital management show that patient safety is top priority	18 (14.9)	34 (28.1)	69 (57.0)	
F9 Hospital management seems interested in patient safety only after an adverse event happens	42 (34.7)	25 (20.7)	54 (44.6)	Needs improvement
F10 Hospital units work well together to provide the best care for patients	8 (6.7)	27 (22.5)	85 (70.9)	
F11 Shift changes are problematic for patients in the hospital	33 (27.3)	27 (22.3)	61 (50.4)	

The composite that has the lowest average percentage positive score was *non-punitive response to error*, at 17%. This composite, along with *communication openness*, *feedback and communication about error*, *frequency of events reported*, *handoffs and transition* and *staffing* was perceived by the HCWs as having weak safety culture (composite score ≤ 50%) and therefore need improvement. Out of the 12 composites, *teamwork within units* has the highest average percentage positive score and was the only area of demonstrable strength (composite score ˃75%), with a score of 79.6% (Table 3) The derived overall average composite score was 47%, indicating that overall the HCWs have a weak perception of safety culture in the hospital, necessitating an improvement.

**Table 3:** Patient safety culture composite scores

Composite	Component items	Positive responses		Total responses		% Positive response to item
Overall perception of patient safety	A10 A15 A17 A18	67 62 55 64		120 119 120 121		67/120 = 55.8% 62/119 = 52.1% 55/120 = 45.8% 64/121 = 52.9%
	Average composite score = 51.7%					
Communication openness	C2 C4 C6	59 12 27		120 118 119		59/120 = 49.2% 12/118 = 10.2% 27/119 = 22.7%
	Average composite score = 27.4% (Needs improvement)					
Feedback and communication about error	C1 C3 C5	33 63 69		121 121 117		33/121 = 27.2% 63/121 = 52.1% 69/117 = 59.0%
	Average composite score = 46.1% (Needs improvement)					
Frequency of events reported	D1 D2 D3	32 20 38		121 121 120		32/121 = 26.4% 20/121 = 16.5% 38/120 = 31.7%
	Average composite score = 24.9% (Needs improvement)					
Handoffs and transition	F3 F5 F7 F11	51 61 53 61		122 120 120 121		51/122 = 41.8% 61/120 = 50.8% 53/120 = 44.2% 61/121 = 50.4%
	Average composite score = 46.8% (Needs improvement)					
Management support for patient safety	F1 F8 F9	60 69 54		122 121 121		60/122 = 49.2% 69/121 = 57.0% 54/121 = 44.6%
	Average composite score = 50.3%					
Non-punitive response to error	A8 A12 A16	22 22 17		121 118 120		22/121 = 18.2% 22/118 = 18.6% 17/120 = 14.2%
	Average composite score = 17% (Needs improvement)					
Organizational learning-Continuous improvement	A6 A9 A13	100 62 69		121 119 120		100/121 = 82.6% 62/119 = 52.1% 69/120 = 57.5%
	Average composite score = 64.1%					
Staffing	A2 A5 A7 A14	17 14 83 24		122 121 121 120		17/122 = 14.0% 14/121 = 11.6% 83/121 = 68.6% 24/120 = 20.0%
	Average composite score = 28.6% (Needs improvement)					
Supervisor/ Manager actions promoting patient safety	B1 B2 B3 B4	90 85 59 99		122 121 122 122		90/122 = 73.8% 85/121 = 70.2% 59/122 = 48.4% 99/122 = 81.1%
	Average composite score = 68.4%					
Teamwork across units	F2 F4 F6 F10	54 73 71 85		122 122 120 120		54/122 = 44.3% 73/122 = 59.8% 71/120 = 59.1% 85/120 = 70.8%
	Average composite score = 58.5%					
Teamwork within units	A1 A3 A4 A11		112 103 92 81		122 122 122 121	112/122 = 91.8% 103/122 = 84.4% 92/122 = 75.4% 81/121 = 66.9%
	Average composite score = 79.6% (Strength)					

In this study the professional roles of the surgeon, nurse anaesthetist and perioperative nurse, correspond with the work areas of surgery, anesthesiology and perioperative nursing, respectively. There were significant differences between the various work areas/ professional groups perception of safety culture in as many as seven out of the twelve composites/dimensions; with the nurse anaesthetists having the highest perception of safety culture and the surgeons having the least (Table 4). Tukey HSD post-hoc test for inter-group comparability further highlighted the relative position of the various professional roles regarding the respective composites (Table 4). The perception of safety culture by the various work areas/professional groups regarding the other five composites was similar.

**Table 4:** **Comparison of patient safety composite scores of the different groups of OR personnel**

Variable	Nurse anesthetists (n = 27) Mean ± SD	Surgeons (n = 59) Mean ± SD	Peri-op nurses (n = 36) Mean ± SD	P value	Tukey HSD post-hoc test (p < 0.05)
Overall perception of patient safety	3.2 ± 0.8	3.1 ± 0.8	3.4 ± 0.7	0.175	
Communication openness	3.0 ± 0.6	2.8 ± 0.8	2.7 ± 0.6	0.199	
Feedback and communication about error	**3.8 ± 0.6**	3.2 ± 0.7	3.2 ± 0.8	0.001	Nurse anaesthetists ˃ periop nurses, surgeons
Frequency of events reported	3.2 ± 0.9	2.6 ± 0.9	**3.0 ± 0.9**	0.023	Nurse anaesthetists ˃ surgeons
Handoffs and transition	**3.4 ± 0.8**	2.9 ± 0.8	3.4 ± 0.7	< 0.001	Nurse anaesthetists, periop nurses ˃ surgeons
Management support for patient safety	**3.6 ± 0.7**	3.1 ± 0.9	3.5 ± 0.9	0.011	Nurse anaesthetists, periop nurses ˃ surgeons
Non-punitive response to error	2.4 ± 0.6	2.5 ± 0.7	2.3 ± 0.7	0.193	
Organizational learning- Continuous improvement	**4.0 ± 0.5**	3.4 ± 0.7	3.6 ± 0.6	0.001	Nurse anaesthetists ˃ surgeons
Staffing	2.6 ± 0.5	2.5 ± 0.7	2.6 ± 0.5	0.403	
Supervisor/ Manager actions promoting patient safety	**4.0 ± 0.4**	3.6 ± 0.7	3.7 ± 0.7	0.035	Nurse anaesthetists ˃ surgeons
Teamwork across units	3.6 ± 0.7	3.3 ± 0.7	3.6 ± 0.6	0.064	
Teamwork within units	**4.1 ± 0.5**	3.7 ± 0.7	3.9 ± 0.6	0.006	Nurse anaesthetists ˃ surgeons
**Group mean composite score**	**3.5 ± 0.3**	3.1 ± 0.5	3.3 ± 0.4	0.003	Nurse anaesthetists ˃ surgeons

As much as 85.2% of the respondents (104/122) did not report any adverse event in the past 12 months (Table 5). Only 38.2% of the respondents (44/115) regarded the patient safety quality in their own work area as very good or excellent (Table 5).

**Table 5:** **Respondent’s grading of patient safety in their respective work area and number of adverse events reported during the last 12 months**

Variable	Frequency	Percent (%)
**In past 12 months, number of EVENT REPORTS made**	**(n = 122)**	
None	104	85.2
1–2 event reports	12	9.8
3–5 event reports	3	2.5
6–10 event reports	3	2.5
**Respondent’s grading of patient safety in their respective work areas**	**(n = 115)**	
**Excellent**	9	7.8
**Very Good**	35	30.4
**Acceptable**	56	48.7
Poor	15	13.0

**Fig. 1:**
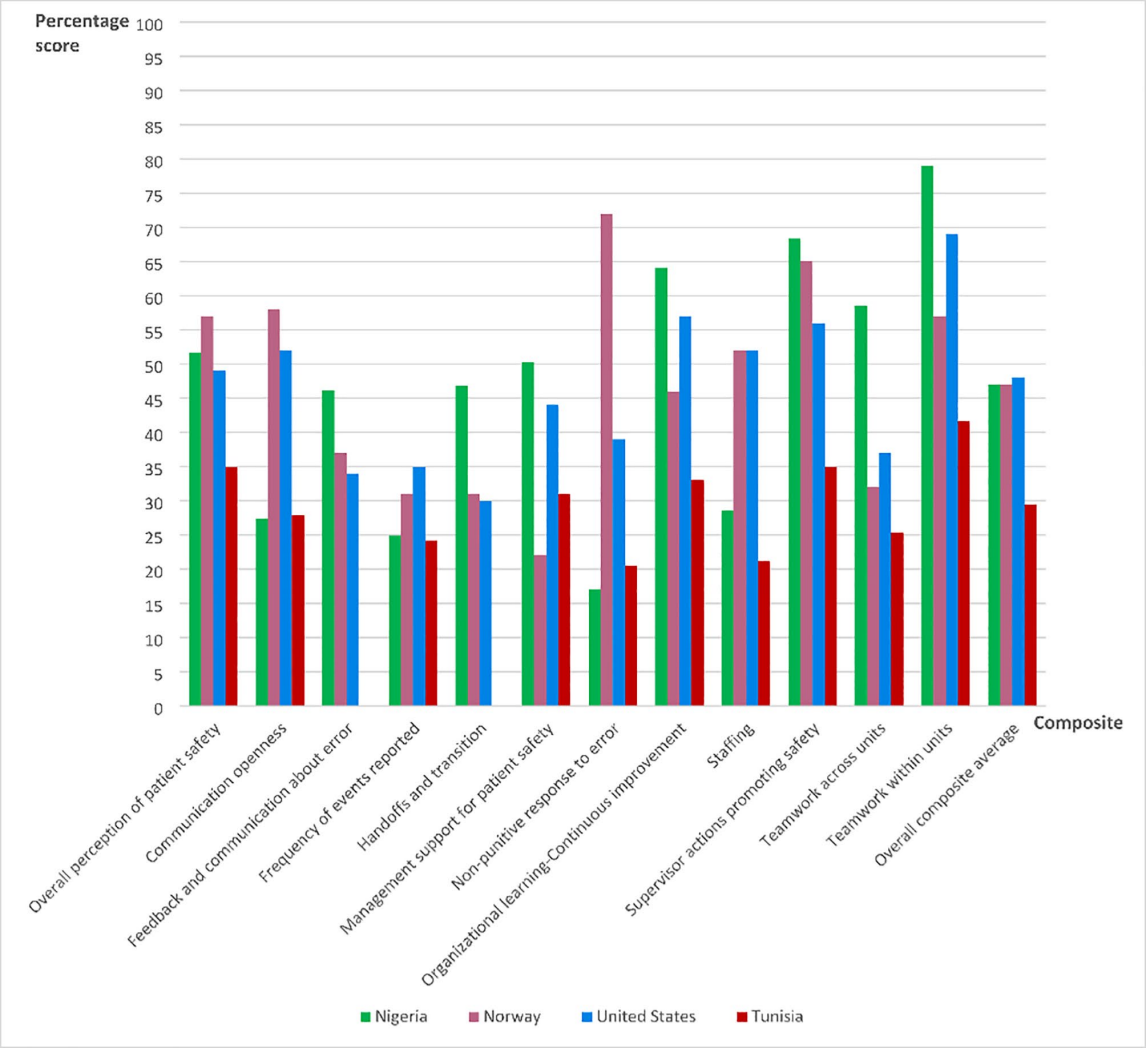
Bar chart showing the composite scores of patient safety culture in our ORs with others from different countries [12, 22, 23]

Figure 1 summarizes the percentage scores of the patient safety composites and overall average composite score in our operating rooms, alongside others from different countries where the HSOPSC instrument was used for safety culture assessment.

The comparative surveys were conducted among OR personnel in five Tunisian hospitals [12] and a single-center survey each in Norway [22] and the United States [23]. The overall average composite score of patient safety culture in our ORs was 47%, compared to the ORs in Tunisia; 29.5%, Norway; 47% and the United States; 48% (Fig. 1). Our ORs had the lowest scores in the dimensions of “*non-punitive response to error*”; 17% and c*ommunication openness*”; 27.4% compared to the ORs in Tunisia, Norway and the United States.

## Discussion

This study investigated the safety culture in the operating rooms of a Nigerian referral hospital using the HSOPSC. The overall average composite was 47%, but dimension scores ranged from 17% for *non-punitive response to error* to 79.6% for *teamwork within units*.

Very few publications have evaluated the patient safety culture in the operating room, among them a Tunisian multicenter study [12], a Norwegian single- institution survey [22] and an American single-institution survey [23]. A few others utilized survey tools other than the HSOPS [24–26] but comparisons with these are not tenable owing to differences in the factor components.

The high response rate of 92.4% obtained in our study may in part derive from the paper-based mode of the survey, single-site location and the size of the sample population. It compares well with the 70.8% response rate of the paper-based multicenter survey that targeted 544 OR staff in five Tunisian hospitals [12]. The Norwegian survey that utilized the mixed distribution method of survey (web and paper modes) in assessing patient safety culture among 575 OR staff had reported a response rate of 62% [22], whereas the online survey that evaluated patient safety culture among 431 OR staff in a United States hospital recorded a response rate of 67% [23]. Paper-based surveys yield higher response rates compared to web-based surveys making them less prone to non-response bias and more reflective of the sample population [27, 28].

The overall average composite score of patient safety culture in our ORs compares well with that of operating rooms in Norway [22] and the United States [23], but exceeds that in Tunisia [12]. Nigeria currently has no national policy on patient safety. However, the implementation of the WHO Surgical Safety Checklist (SSC) in our hospital since 2013 may have impacted on the safety culture despite the obvious constraints of infrastructure and socioeconomic limitations in our environment. Kawano et al. had earlier documented the positive effect of WHO SSC implementation by surgical teams on safety attitudes and climate in the hospital setting in Japan [29]. With the implementation of a National Patient Safety Campaign in Norway (2011–2013) which has SSC compliance rates at hospital level as a quality indicator, a longitudinal cross-sectional study was conducted in a large Norwegian tertiary hospital to evaluate its impact on safety culture by comparing the pre- and post-intervention safety culture perception among OR personnel [30]. Their study revealed that introduction of the WHO SSC brought about improvement in all the patient safety culture composites and that compliance rate in the use of the SSC correlated positively with improvements in safety culture composites/dimensions. We observed wide disparity across the patient safety composites, being highest in *teamwork within hospital units* and lowest in *non-punitive response to error*. *Teamwork within hospital units* defines the extent to which the respondents in a unit support each other, treat each other with respect, and work together as a team. This composite was the strongest in our study as well as the studies conducted in the United States [23, 31] and Tunisia [12]. *Non-punitive response to error* defines the extent to which the respondents feel that their mistakes and event reports are not held against them and that mistakes are not kept in their personnel record. The very low score is indicative of a prevalent culture of blame. Blame, and the fear of blame have been recognized as constituting pernicious impediment to patient safety as they are associated with lack of trust and poor reporting culture [32]. The poorest perception attributed to this safety culture composite in our study was shared by other studies [12, 31] but contrasts sharply with the Norwegian ORs where it was the strongest composite [22]. The high score of this composite in the Norwegian ORs could be reflective of an enduring “system approach” as against the more prevalent “person approach” to error management [32].

The very low perception regarding *non-punitive response to error* in our ORs correspond with an equally low tally of *frequency of events reported*; both signifying a poor reporting culture. Compared to the ORs in Tunisia, Norway and the United States, our personnel had better perception of seven patient safety composites regarding; *teamwork within units*, *teamwork across units*, *organizational learning-continuous improvement*, *management support for patient safety*, *supervisor actions promoting safety*, *handoffs and transition*, and *feedback and communication about error*. None of the three considered studies in previous literature could pride itself of any area of strength with respect to the 12 patient safety dimensions [12, 22, 23]. Equally dismal were the findings of a recent survey of five cardiovascular surgical centers in the United States, further alluding to pervasive poor safety culture in the ORs [33].

The safety culture perception in our ORs is comparable, and arguably better, than that in ORs cited in Norway and the United States despite the huge socioeconomic disparities. It would thus appear that factors beyond the socioeconomic milieu, such as the implementation of the WHO Surgical Safety Checklist (SSC) in our hospital may have played a positive role in the perception of patient safety culture by the OR personnel. The impact of such protocol implementation on safety attitudes is documented, and has been stated earlier [29]. Thus, even resource-poor environments could have better or comparable patient safety culture with those that have economic advantage.

In the light of suggestions that variations exist in the perception of safety culture by HCWs with different professional backgrounds [33], we conducted a subanalysis of the responses based on professional roles and work areas. We observed that the various categories of personnel in the OR rated safety culture differently. Our finding was supported by the Norwegian study wherein anesthesiologists and nurse anaesthetists had higher mean scores than the surgeons and operating theatre nurses [22]. Similarly, the Tunisian study reported that physicians rated the safety culture of operating rooms lower than the paramedical staff (nurses, anaesthetic and surgical technicians, nurses’ assistants) in most of the dimensions [12]. The lowest perception of patient safety among the surgeons implies that they were the least optimistic of the existing safety culture. An international survey on safety culture and attitudes among spine professionals had earlier revealed that most of the respondents believe that the surgeon has responsibility for both the prevention of adverse events and improvement of the safety culture in the operating room [25]. Such a mindset could influence a more critical appraisal of patient safety among the surgeons compared to the perioperative nurses and the nurse anaesthetists. Remarkable variation in perception between the different categories of personnel had also been reported by studies on safety climate conducted with the SAQ among OR personnel in Brazil and Sweden [34, 35].

As much as 85% of our respondents made no report of adverse events over the past 12 months. The Tunisian study which presented data on adverse event reporting also declared that 90.2% of the respondents had reported no adverse event in the past 12 months [12]. It is likely that events were underreported in these settings and several potential patient safety problems may not have been recognized and addressed, posing further danger to the patients.

The systems approach has been recommended and effectively implemented in error management in high risk industries like aviation [36]. However, its application in healthcare is still constrained while the persisting ‘culture of blame’ propels wanton administrative, professional and legal liabilities on HCWs for medical errors [37]. This culture arguably contributes to the festering poor scores in the dimensions of *non-punitive response to error* and *frequency of adverse events reported*. Furthermore, the decision to report errors by HCWs is influenced by their proneness to shame and their perception of the organizational attitude towards restoring their self-image [38]. This would suggest that both *non-punitive response to errors* and *management support for patient safety* would enhance error reporting which is a crucial process in medical error management and patient safety.

The 2021 User Comparative Database Report for Version 1.0 obtained from 191,977 hospital staff in 320 hospitals in the United States who were surveyed between December 2017 and.

October 2020, recorded a much better ‘overall average composite score’ of 65% compared to the 47% of our study [31]. But a major flaw in comparing OR patient safety culture reports with the 2021 User Comparative Database Report for Version 1.0 of the United States derives from the fact that the latter surveyed hospital-wide personnel encompassing administrative staff, rehabilitation, medicine, pharmacy, et cetera. Interestingly, respondents in the work areas of anesthesiology and surgery constituted only 1% and 11% of the surveyed population, respectively; suggesting that the majority of the respondents were non-OR personnel. With such differing characteristics in work area and staff position the perception of the respondents in the latter could not be adjudged to represent the culture of the OR environment in view of known professional and work area-related disparities in safety culture perception. For instance, the work area characteristics of the 2021 User Comparative Database Report revealed that respondents from rehabilitation section (work area) had the highest average composite score of 72%, while respondents from administration (staff position) had the highest average composite score of 78%. These were much higher than the composite scores attributed to anesthesiology, surgery, attending surgeons, residents and registered nurses who characterize the OR environment. Moreover, as much as 22% of the respondents in the comparative database do not have any direct interaction with the patients.

Several broad-based initiatives have been embarked upon by governments to systemically and specifically improve patient safety. In Sweden patient safety got a boost in 2011 with the enactment of the patient safety act and implementation of government-supported financial incentive for patient safety actions in healthcare facilities, including safety culture improvement [39]. In Denmark too, the legislation on patient safety was passed in 2003 and sought to improve patient safety by; ensuring that (i) frontline personnel report all adverse events (ii) hospital acts on the reports (iii) the National Board of Health disseminate learning from them while protecting the personnel from disciplinary investigations and legal sanctions [40]. In aligning with the above initiatives, ‘improvement in organizational culture to encourage reporting and avoid blame’ received the strongest recommendation of 22 suggested options for enhancing patient safety by Swedish patient safety-oriented healthcare professionals, whereas increasing the number of physicians, nurses, and hospital beds were rated 12th, 15th and 17th respectively and ‘increased penalty for personnel who make mistakes’ got the least recommendation [39].

The HSOPSC survey, like the other patient safety instruments measure abstract phenomena termed composite/ dimensions from self-reported perceptions of safety culture and attitudes. Such models facilitate data reduction by means of orderly simplification of a number of interrelated measures. The use of instruments with sound psychometric property is thus critical since the multiple items measured are presumed to represent the fewer underlying constructs. The HSOPSC has been validated in over 62 studies conducted in over 29 countries [41]. However, in spite of its popularity and wide application its psychometric properties have been challenged, with some researchers advocating revision of some of the instrument’s items and composites [22, 41]. A revision of the original Hospital Survey on Patient Safety Culture version 1.0 survey has recently been released by AHRQ (HSOPS 2.0) [42].

Furthermore, the survey being a self-reported perception the potential for response bias cannot be ruled out. Our assessment of patient safety culture in the OR was not comprehensive, as the very few number of physician anaesthetists precluded them from the study (in line with the instrument guideline), while health attendants were not considered owing to their poor comprehension of the instrument. Nevertheless, the surveyed personnel represent over 85% of the OR staff and could justifiably be deemed representative.

Despite having the English language as the lingua franca in Nigeria, we did not conduct cultural adaptation and further validation of the original English version of the HSOPSC which was developed within the American cultural environment in order to ensure the equivalence of meaning for this cross-cultural research as it were. Hence, whereas we did not substantially alter the original validated English version which would contribute to psychometric distortions the results we obtained may not have accurately reflected what they are supposed to measure. Thus, we concede that the use of a previously validated instrument does not necessarily imply validity in another culture or context [43, 44]. However, such limitations that may arise from variations in the psychometric properties of measurement instruments are a common feature in cross-cultural research, including those conducted with the HSOPSC [45]. It must also be acknowledged that the multiplicity of other factor models proposed for the HSOPSC instrument in different studies such as the 11- factor [22], 10-factor [46], 9-factor [47] and 8-factor models [48] complicate the process of comparing outcomes. Our study instituted only a minor modification of item F3 by rewording it as indicated in the methods section; a minimum which is permissible by the instrument developers [14]. The French validated version of the Hospital Survey on Patient Safety Culture questionnaire used by Mallouli et al. comprised only of 10 composites with 45 items [12], as against 10 composites and 42 items in the original version which we used. In view of such variations which are rather common with the different adaptations of the instrument direct comparison of the results of different surveys demands circumspection.

### Recommendations

It is hoped that the implementation of relevant interventions that this study has spurred will bring about improvement in the safety culture of our ORs, as have been observed in follow-up studies conducted in Saudi Arabia [49], Japan [29] and Norway [30]. Furthermore, with this benchmark a follow-up survey to evaluate the outcome of implemented interventions would be necessary, and is highly recommended.

## Conclusion

So far, despite subtle variations in the versions of the HSOPSC questionnaire used in the different studies, our study appears to be the first to record even one area of strength across the composites of patient safety culture. With a low overall average composite score and as many as half of the composites requiring improvement our OR safety culture could be adjudged to be weak, its comparative strengths notwithstanding. The finding is disconcerting owing to the association between weak patient safety climate and poor patient outcomes. The picture of safety culture emanating from the ORs discussed herein is worrisome and may indeed be a major contributor to the gloomy statistics of surgery-related morbidity and mortality, globally.

## Data Availability

All data generated or analyzed during this study are included in this published article.

## Abbreviations

AHRQ: Agency of Healthcare Research and Quality
ANOVA: Analysis of variance
HCW: Healthcare worker
HSOPSC: Hospital survey on patient safety culture
INAES: Irish National Adverse Events Study
OR: Operating room
SAQ: Safety attitudes questionnaire
SPSS: Statistical package for social science
SSC: Surgical safety checklist
WHO: World health organization
